# Data report on gene expression after hepatic portal vein ligation (PVL) in rats

**DOI:** 10.3389/fgene.2024.1421955

**Published:** 2024-08-21

**Authors:** Daria Meyer, Joanna Kosacka, Martin von Bergen, Bruno Christ, Manja Marz

**Affiliations:** ^1^ Bioinformatics and High-Throughput Analysis, Friedrich Schiller University Jena, Jena, Germany; ^2^ Oncgnostics GmbH, Jena, Germany; ^3^ Cell Transplantation/Molecular Hepatology Lab, Department of Visceral, Transplant, Thoracic, and Vascular Surgery, University of Leipzig Medical Center, Leipzig, Germany; ^4^ Molecular Systems Biology, Helmholtz Centre for Environmental Research–UFZ, Leipzig, Germany; ^5^ FLI Leibniz Institute for Age Research, Jena, Germany; ^6^ European Virus Bioinformatics Center, Jena, Germany; ^7^ Michael Stifel Center Jena, Jena, Germany; ^8^ Aging Research Center (ARC), Jena, Germany; ^9^ German Centre for Integrative Biodiversity Research (iDiv) Halle-Jena-Leipzig, Leipzig, Germany; ^10^ Max Planck Institute for the Science of Human History, Jena, Germany

**Keywords:** portal vein ligation, PVL, rat liver, liver, RNA-seq, gene expression, transcriptomics

## 1 Introduction

Due to the increasing life expectancy and life style, the incidence of primary liver cancer is steadily rising. Worldwide, it is the fourth leading reason of cancer-associated death (Yang et al., 2019; Sung et al., 2021). The etiology of liver cancer is highly diverse including besides others viral, toxic, nutritional, etc. risk factors that render treatment options as complex as different pathogenic pathways are involved (Forner et al., 2006; Forner et al., 2018; Llovet et al., 2021). Besides novel pharmacological and cell therapy approaches, surgical interventions are the only potentially curative strategies (Angeli-Pahim et al., 2023). Among these, hepatic resection intends to remove the solid tumor taking into account tumor location and size as well as vascular supply of the parts of the liver to be removed and of the remaining liver. Since the lobar organisation of the liver is mirrored by separate venous blood drainage of the lobes, liver resection in general means removal of the tumor bearing lobe(s). The loss of even more than 60% of the liver mass may be tolerated given that the future liver remnant provides sufficient post-surgery regenerative and metabolic function. To enhance the function of the future liver remnant, the technique of portal vein embolisation is used clinically. This procedure aims at increasing the future liver volume anticipating that volume equals function, i.e., volume growth of the non-ligated lobe(s) compensates for the surgical liver mass loss. However, volume does not necessarily reflect function. Therefore, in order to assess post-surgery hepatic metabolic and regenerative capacities, it is necessary to characterise changes in gene expression in the atrophic and hypertrophic lobes, respectively, and to correlate these changes with the prospective functional efficiency of the liver remnant after resection of the tumor bearing ligated liver lobe(s).

**FIGURE 1:**
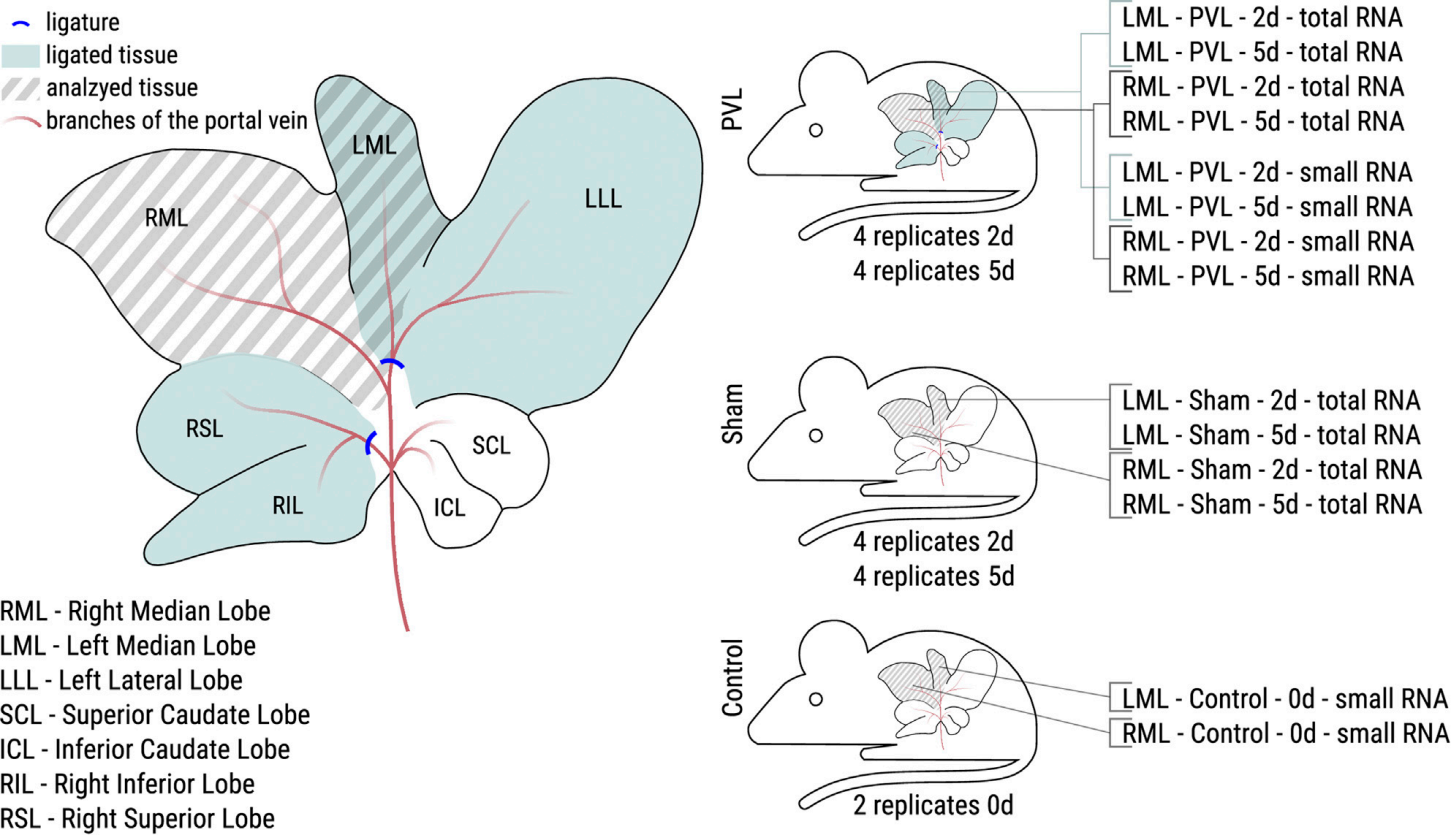
Overview of the performed PVL surgery of the rat liver and the resulting analyzed samples. **Left:** During PVL surgery two portal vein branches were ligated (blue lines). Samples were taken from the right median lobe (RML, non-ligated during PVL) and the left median lobe (LML, ligated during PVL) for all animals. **Right:** PVL and sham surgery were performed for eight animals each. Half of the animals were sacrificed after 2 days (2d), the other half after 5 days (5d). Control animals were not operated at all. For PVL and sham animals, total RNA libraries were made, for PVL and control animals, small RNA libraries, resulting in 32 and 20 samples for total and small RNA, respectively.

Experimentally, portal vein ligation is comparable with portal vein embolisation applied in clinical settings. For the data collection presented here, we applied a model of 60% portal vein ligation in the rat, i.e., the left median and the lateral left lobes as well as the right superior and inferior lobes were deprived by ligation of the portal vein, while the right median and the left superior and inferior lobes remained unaffected (see Figure 1), accordingly modified as described (Sänger et al., 2015; Wei et al., 2020a).

Although non-coding RNAs (ncRNAs) such as microRNA (miRNA) or long ncRNA (lncRNA) have been shown to be deeply involved in the pathophysiology of almost all acute and chronic liver diseases, many of the especially liver-specific ncRNAs, have been not even annotated yet (Roy et al., 2018). Our group developed new transcriptome assembly pipelines by combining existing tools to identify the different isoforms of mRNAs and lncRNAs (Hölzer and Marz, 2019; Faber and Hölzer, 2020).

### 1.1 Non-coding RNAs in human genome

From the pilot project of ENCODE we know that less than 3% of the human genome code for proteins (ENCODE Project Consortium et al., 2007; ENCODE Project Consortium, 2012). The remaining genome is divided into 45% repetitive elements (SINEs, LINEs, transposons), 26% introns and other unique non-coding DNA (Gregory, 2005). The question of their meaning has raised 4 replicates 5d is only being answered slowly. By now, we know that at least 80% of the human genome has a function (ENCODE Project Consortium, 2012). Some ncRNAs are known to be located within introns, in 5′ and 3′ untranslated regions (UTRs), antisense to protein-coding sequences or just close to them. However, the *in silico* identification of the ncRNAs is still a huge challenge. Due to their diverse and fast evolving sequence they are identified most efficiently by a combined *in silico*/*ex vivo* approach, that is sequencing the transcriptome and establishing tissue/organ/organism-specific bioinformatical tools. During the last decade, miRNAs, as regulators of various cell processes, received major attention. However, currently there are 3,016 ncRNA families described [Rfam v.14.1 (Kalvari et al., 2018)], of which 800 are associated to human, covering more than 16.000 genomic regions^1^. Additionally, the existence of long non-coding RNAs ( > 200 nt) containing introns themselves is estimated in humans by GENCODE v.33 to 17,952 loci^2^. Our knowledge about lncRNAs is limited and no general computer program for their identification including secondary structure information and protein interactions is developed, yet.

### 1.2 Non-coding RNAs in liver

Although only a fraction of liver-specific ncRNAs are known, here we summarize important examples of ncRNA participating in the pathogenesis of different forms of liver disease and how they can be used as therapeutic tools or targets for novel treatment paradigms, following the suggestions of Roy et al. (2018), see Table 1. A large number of ncRNA genes being involved in metabolic processes, inflammation and immune response are differentially expressed during aging and other biological processess. We therefore expect to see ncRNAs and especially miRNAs to play a role in senescence and inflammation in rat liver when comparing PVL with healthy liver (Barth et al., 2019).

**TABLE 1:** NcRNAs altered in liver diseases (Roy et al., 2018), and citations in there. HSC–hepatic stellate cells.

ncRNA	Targets	
HSC-specific		
miR-29	IGF-I, PDGF-C, HSP47, Collagens	
miR-30	KLF11	
miR-200	α -SMA, β -catenin, TGF β -2	
miR-122	P4HA1, FN1, SRF	
miR-21	PTEN, API, SPRY2, HNF4, PDCD4	
miR-34a	ACSL1	
MEG3	I κ b α	
MALAT1	CXCL5	
NEAT1	miR-122, KLF-6	
PVT1	miR-152	
GAS5	miR-222	
lincRNA-p21	p21	
Hepatocyte-specific		
miR-122	CyclinGl, ADAM10, IGF1R, SRF	
miR-192	Zebl, Zeb2	
Hand2	C-met	
00321	CyclinBl	
ARSR	Akt/SREBP-lc	
HULC	miR-186	
Kupffer cells-specific		
miR-155	Smad3, C/EBP β	
miR-223	Caspase 3	
Cholangiocyte-specific		
miR-124	STAT3, IL-6R	
H19	SHP	

### 1.3 MicroRNAs in liver

MicroRNAs (miRNAs) are a class of small, non-protein coding RNAs that play a crucial role in mediating post-transcriptional gene silencing (Carthew and Sontheimer, 2009). These molecules, typically 18 to 25 nucleotides in length, act as regulators of gene expression by binding to the 3′ untranslated region of target genes (Bartel, 2009). Interestingly, the intricate machinery governing miRNAs holds significant importance not only in the context of liver diseases (Szabo and Bala, 2013), but also in processes related to portal vein ligation (Song et al., 2010). In the realm of liver biology, miRNAs exert profound effects on various aspects of liver function and pathology. They participate in regulating processes such as hepatocyte proliferation (Song et al., 2010), differentiation, apoptosis, and lipid metabolism (Agbu and Carthew, 2021). Dysregulation of miRNAs has been implicated in the pathogenesis of liver diseases ranging from viral hepatitis (Shrivastava et al., 2015) to hepatocellular carcinoma (Nagy et al., 2018). Moreover, the role of miRNAs in portal vein ligation, a surgical procedure often used in experimental models to study liver regeneration, underscores their significance beyond disease states (Starlinger et al., 2019). MiRNAs are intricately involved in the molecular pathways underlying the response to portal vein ligation, influencing the regeneration capacity of the liver and impacting overall hepatic function (Chen et al., 2017). Overall, the multifaceted involvement of miRNAs in liver biology and portal vein ligation highlights their versatility and potential as therapeutic targets in the context of liver diseases and surgical interventions.

## 2 Materials and methods

### 2.1 Animals and sample collection

Experiments were conducted from March 1 until April 13, 2022. Male Sprague-Dawley rats (Charles river, 320–440 g) were housed under a 12 h dark/light cycle at ambient temperature with free access to food and water. The experiments were run with four different groups including four animals each for the PVL and the sham operation at two different time points (2 and 5 days) after surgery (16 animals). An additional weight-matched two animals without any treatment were run along as control in order to identify potential surgery-induced differences as compared with the sham-operated animals. The two time points were chosen following the rational that post-surgery regeneration after PVL might follow similar kinetics as regeneration after partial hepatecomy featuring a maximum regenerative response after 2 days and returning to starting conditions again after 5 days (Gerlach et al., 1997; Andersen et al., 2013). The PVL operation was a modified procedure as described previously (Wei et al., 2020b) by ligating in addition to the left median and left lateral portal vein before the bifurcation, the right superior and inferior portal veins before the bifurcation. In sham-treated animals, the abdominal cavity was opened by a midline incision and closed again thereafter. After the sham or the PVL operation, the animals were left under housing conditions. At 2 and 5 days after surgery, respectively, the animals were euthanized under 2% isoflurane anesthesia and livers explanted. The weight-matched control animals without any treatment were run along. Tissue pieces (ca. 50 mg) from the left (LML, ligated) and the right (RML, non-ligated) median lobes (Figure 1) were immediately collected in 750 µL QIAzol reagent (Quiagen GmbH, Hilden, Germany) in 2 mL reaction tubes (Biozym Scientific GmbH, Hessisch Oldendorf, Germany) containing 5 PreCelly beads (PEQLAB Biotechnologie GmbH, Erlangen, Germany), snap frozen and stored at −80°C until further analysis. In total, 52 samples from 18 animals were passed to RNA extraction and subsequent sequencing.

### 2.2 RNA isolation

Frozen samples were thawed and centrifuged twice for 10 s at 5,500 rpm in the PRECELLYS^®^ 24 homogenizer (VWR International GmbH, Darmstadt; Germany), and mixed with 150 µL chloroform for 30 s using a vortexer. After incubation for 10 min on crashed ice, phases were separated by centrifugation at 4°C for 15 min and 11,000 rpm in the Fresco 21 centrifuge (Haereus, Hanau, Germany). The aqueous phase was collected, the RNA precipitated with 500 µL isopropanol overnight at −20°C and subsequently centrifuged for 10 min at 4°C in the benchtop microcentrifuge. The pellet was washed with 500 µL 3 M sodium acetate and the suspension pelleted for 10 min at 4°C and 11,000 rpm. This step was repeated, the final pellet dissolved in 300 µL RNA grade water at 4°C for 2 h. Thereafter, the RNA was precipitated with 600 µL ethanol abs. at −80°C for 1 h and pelleted for 10 min at 4°C and 11,000 rpm. The pellet was washed with 500 µL 80% ethanol and the pellet collected for 10 min at 4°C and 11,000 rpm. After repetition of this step, the final pellet was collected and shortly air dried. The RNA was dissolved in 75–100 µL of RNA grade water overnight in the fridge. The RNA content and quality were determined using the Amersham NanoVue equipment (GE Healthcare, Freiburg, Germany).

### 2.3 Sequencing

Total cellular RNA from the tissues were isolated. cDNA libraries were prepared for all 16 operated animals, utilizing the NEBNext Ultra II Directional RNA library preparation kit including rRNA depletion (rRNA-) according manufactures instructions. The complete experiment was performed in four independent, biological replicates; thus, 32 rRNA-libraries were sequenced on a NovaSeq6000 run yielding 251 bp reads on paired-end mode (further referred to as “total RNA dataset”). For all animals which underwent a PVL surgery, additional cDNA libraries were prepared utilizing the TruSeq Small RNA library preparation kit according manufactures instructions, to sequence especially small RNAs. Additionally, for the two control animals, cDNA libraries were prepared utilizing the TruSeq Small RNA library preparation kit according manufactures instructions. The resulting 20 libraries were sequenced on a NovaSeq6000 run yielding 51 bp reads on paired-end mode (further referred to as “small RNA dataset”).

### 2.4 Data preprocessing: trimming, mapping and differential gene expression analysis

The sequencing data was quality controlled using fastQC (v0.11.9)^3^. Adapter sequences and low quality bases were removed by trimmimg with fastp (v0.20.1) (Chen et al., 2018). Parameters for total RNA dataset: --detect_adapter_for_pe --length_required 20 --cut_right --cut_mean_quality 28; parameters for small RNA dataset: --adapter_fasta --cut_right --length_required 15 --cut_mean_quality 28; Parameters for adapter file see Supplementary Data Sheet 1.FASTA, then quality was controlled again using fastQC (v0.11.9).

Mapping and counting of reads were performed differently for the total and the small RNA dataset. For the total RNA dataset, the reads were mapped with HISAT2 (v2.2.1) (Kim et al., 2015) using default parameters to the *R. Norvegicus* reference genome mRatBN7.2 (release 108, retrieved on 11/09/2022 from Ensembl^4^). Data was converted into sorted bam files using samtools (v1.12) (Li et al., 2009) and reads per gene were counted using featureCounts (v2.0.1) (Liao et al., 2014) using the reference annotation mRatBN7.2 (release 108, retrieved on 11/09/2022 from Ensembl^5^) including multimapping reads (featureCounts -M -p -T 20 -t ’exon’). For the small RNA dataset, the reads were mapped with HISAT2 (v2.2.1) (Kim et al., 2015) using default parameters to the *R. norvegicus* reference genome mRatBN7.2 (release 108, assembly accession GCF_015227675.2, retrieved on 06/01/2023 from NCBI^6^). Data was converted into sorted bam files using samtools (v1.12) (Li et al., 2009). For counting, a miRNA specific annotation file was generated, based on the NCBI assembly GCF_015227675.2 for mRatBN7.2 (retrieved on 06/01/2023 from NCBI^7^). The created miRNA annotation file contains a 5′ and 3′ miRNA entry for each miRNA, either taken from the original annotation or generated by splitting the pre-miRNA entry for the miRNA into two-halves (see Supplementary Data Sheet 3.csv). The reads per miRNA were counted using featureCounts (v2.0.1) (Liao et al., 2014) including multimapping reads (featureCounts -M -p -T 20 -t ’exon’).

Sequencing statistics (see Tables 2, 3) were extracted from fastp report files (amount of reads, read length) and from featureCounts summary file. We used the percentage of reads assigned to annotated features and percentage of reads, which could not be (unambiguously) assigned to features, or not mapped to the reference at all. The percentage of risobosomal RNA (rRNA) was calculated using sortMeRNA (v4.3.6) (Kopylova et al., 2012) with the default sortMeRNA reference database v4.3.4 (smr_v4.3_default_db.fasta).

**TABLE 2:** Sequencing statistics for total RNA (NEBNext Ultra II Directional RNA).

Sample	RNA	After Sequencing				After Trimming			After Mapping			
	ng/µl	Reads	RL R1	RL R2	% dup	Reads	RL R1	RL R2	% MF	% MG	% UA	% rib
PVL												
2d-LML-01	300	24.85	251	251	13.68	23.4	183	174	60.60	35.76	3.64	47.55
2d-LML-02	300	23.88	251	251	14.00	22.4	184	173	52.32	44.51	3.17	44.28
2d-LML-03	300	25.79	251	251	13.57	24.1	188	176	58.55	38.05	3.40	41.18
2d-LML-04	300	23.90	251	251	13.86	22.4	187	176	55.32	41.19	3.49	42.53
5d-LML-01	300	24.26	251	251	13.65	22.7	188	176	58.94	37.71	3.35	40.29
5d-LML-02	300	23.14	251	251	13.66	21.6	185	174	57.07	39.62	3.31	42.83
5d-LML-03	300	20.85	251	251	13.49	19.5	188	177	61.39	35.09	3.53	40.81
5d-LML-04	300	19.44	251	251	13.77	18.2	183	173	57.33	39.43	3.25	45.49
2d-RML-01	300	26.92	251	251	13.79	25.4	175	167	59.02	36.99	3.99	58.00
2d-RML-02	300	18.16	251	251	12.88	17.0	187	176	60.07	36.48	3.45	42.53
2d-RML-03	300	24.61	251	251	13.55	23.0	187	176	60.78	35.71	3.52	43.60
2d-RML-04	300	19.48	251	251	13.85	18.3	185	175	55.43	41.04	3.53	45.57
5d-RML-01	300	25.16	251	251	13.45	23.5	187	176	61.24	35.30	3.47	42.45
5d-RML-02	300	24.84	251	251	13.35	23.3	186	175	61.12	35.42	3.46	43.08
5d-RML-03	300	24.49	251	251	13.47	22.9	193	180	60.45	36.08	3.48	36.08
5d-RML-04	300	21.69	251	251	13.80	20.6	179	174	60.41	36.34	3.24	49.80
SHAM												
2d-LML-01	300	23.93	251	251	13.24	22.5	180	171	61.84	34.26	3.90	50.37
2d-LML-02	300	24.56	251	251	14.04	22.9	187	176	60.43	35.91	3.67	43.22
2d-LML-03	300	27.71	251	251	13.13	26.0	183	173	60.19	36.26	3.56	46.06
2d-LML-04	300	25.44	251	251	13.67	23.9	183	172	59.55	36.84	3.61	47.44
5d-LML-01	300	24.78	251	251	14.16	23.2	186	176	57.58	37.61	4.81	44.26
5d-LML-02	300	24.25	251	251	13.66	22.8	180	171	61.58	34.58	3.84	51.97
5d-LML-03	300	26.30	251	251	13.13	24.7	184	175	59.66	36.53	3.82	46.44
5d-LML-04	300	25.08	251	251	13.09	23.5	182	174	58.60	36.90	4.50	46.80
2d-RML-01	300	27.78	251	251	13.79	26.1	184	173	61.98	34.02	4.00	48.00
2d-RML-02	300	23.49	251	251	13.25	22.0	184	173	60.55	35.71	3.74	45.85
2d-RML-03	300	29.37	251	251	13.55	27.5	182	172	59.63	36.48	3.89	43.92
2d-RML-04	300	27.51	251	251	13.82	25.8	186	175	59.41	36.97	3.61	42.93
5d-RML-01	300	27.11	251	251	13.63	25.4	183	173	60.44	35.22	4.34	47.35
5d-RML-02	300	25.58	251	251	14.07	24.0	184	174	60.39	36.09	3.52	45.18
5d-RML-03	300	27.13	251	251	14.31	25.5	182	171	59.48	36.78	3.73	47.32
5d-RML-04	300	23.44	251	251	14.18	21.8	184	172	60.11	36.32	3.57	43.77

PVL, animals underwent PVL surgery; sham, animals underwent sham surgery; 2d, 5d, animals sacrificed 2 or 5 days after surgery, respectively; RML, right median liver lobe; LML, left median liver lobe; RNA, RNA concentration after extraction; Reads (10^6^) and mean read length (RL of paired reads R1 and R2) extracted from fastp reports, regarding only priamry alignments; dup, read duplication rate extracted from fastp; MF, mapped reads (incl. multimapping) onto annotation; MG, mapped reads (incl. multimapping) onto unannotated regions; UA, unmapped and ambigiously assigned reads; MF, MG, UA were extracted from featureCounts; rib, % mapped to rRNA by sortMeRNA.

**TABLE 3:** Sequencing statistics for small RNA (TruSeq Small RNA).

Sample	RNA	After Sequencing				After trimming			After mapping			
	ng/µl	Reads	RL R1	RL R2	% dup	Reads	RL R1	RL R2	% MF	% MG	% UA	% rib
PVL												
2d-LML-01	300	74.28	51	51	46.50	65.23	23	23	61.27	15.58	23.16	1.36
2d-LML-02	300	119.16	51	51	37.79	100.92	24	24	67.60	14.58	17.82	2.52
2d-LML-03	300	79.45	51	51	42.98	70.09	24	24	61.41	16.03	22.56	1.58
2d-LML-04	300	99.20	51	51	45.78	87.47	23	24	61.63	15.49	22.89	1.72
5d-LML-01	300	75.68	51	51	43.94	66.04	24	24	58.37	19.01	22.63	2.69
5d-LML-02	300	106.20	51	51	46.47	93.54	23	23	62.93	13.70	23.37	1.37
5d-LML-03	300	97.61	51	51	48.42	86.80	23	23	63.20	13.34	23.45	1.17
5d-LML-04	300	103.88	51	51	45.39	92.84	23	23	63.56	13.92	22.52	1.43
2d-RML-01	300	81.72	51	51	46.53	71.67	23	23	59.14	14.44	26.41	1.12
2d-RML-02	300	65.91	51	51	45.72	57.51	23	23	61.43	13.86	24.71	0.97
2d-RML-03	300	95.83	51	51	50.85	88.26	23	23	61.60	13.98	24.42	0.98
2d-RML-04	300	85.73	51	51	46.14	78.26	23	23	60.66	16.94	22.41	1.81
5d-RML-01	300	105.18	51	51	50.72	95.76	23	23	61.39	15.92	22.69	1.25
5d-RML-02	300	107.85	51	51	51.95	98.37	23	23	60.37	14.91	24.72	1.13
5d-RML-03	300	81.76	51	51	47.56	74.62	23	24	61.72	15.69	22.59	1.10
5d-RML-04	300	99.53	51	51	50.15	91.40	23	23	63.72	13.86	22.42	0.99
CONTROL												
0w-LML-01	300	84.42	51	51	52.32	78.48	23	23	64.97	11.47	23.56	0.91
0w-LML-02	300	76.46	51	51	52.94	70.97	23	23	64.96	11.83	23.21	0.91
0w-RML-01	300	85.13	51	51	44.82	79.15	23	23	62.42	13.60	23.98	0.86
0w-RML-02	300	91.29	51	51	48.04	85.45	23	23	64.54	12.24	23.22	0.81

PVL, animals underwent PVL surgery; sham, animals underwent sham surgery; 2d, 5d, animals sacrificed 2 or 5 days after surgery, respectively; 0w, animals without treatment; RML, right median liver lobe; LML, left median liver lobe; RNA, RNA concentration after extraction; Reads (10^6^) and mean read length (RL of paired reads R1 and R2) extracted from fastp reports, regarding only priamry alignments; dup, read duplication rate extracted from fastp; MF, mapped reads (incl. multimapping) onto annotation; MG, mapped reads (incl. multimapping) onto unannotated regions; UA, unmapped and ambigiously assigned reads; MF, MG, UA were extracted from featureCounts; rRNA, % mapped to rRNA by sortMeRNA.

## 3 Resulting data

### 3.1 Total RNA

The sequencing of the 32 total RNA samples (see Table 2) resulted in 251 nt paired end reads (R1 and R2), with an average of 24.5 million reads per sample with number of reads per sample ranging from about 18.2 million reads for sample PVL-2d-RML-02 up to about 29.4 million reads for sample sham-2d-RML-03. The read duplication rate ranges from 12.88% in sample 2d-RLM-02 to 14.31% in sample 5d-RML-03, with an average read duplication rate of 13.64%. After trimming low quality bases and removing adapter sequences, the number of reads and the average read length decreased to about 23 million reads per sample and average read length of 184 and 174 nt for R1 and R2 reads, respectively. For all samples, about 60% of the reads could be assigned to annotated features by using featureCounts and including multimapping reads (min. 52%, max. 62%). On average, 36% of the reads could be mapped unambiguously onto the genome into regions with no annotated features, while about 4% of the reads could not be mapped unambiguously onto the rat genome (either reads not mapping to the reference genome at all or reads mapping to multiple features). Up to 58% of the unprocessed reads could be aligned to ribosomal RNA (rRNA), based on the sortMeRNA default database with an average of 45% aligning to rRNA over all total RNA samples.

### 3.2 Small RNA

The sequencing of the 20 small RNA samples (see Table 3) resulted in 51 nt paired end reads (R1 and R2), with an average of 90.81 million reads per sample with number of reads per sample having a wide range from 65.91 million reads for sample PVL-2d-RML-02 up to 119.16 million reads for sample PVL-2d-LML-02. The read duplication rate ranges from 37.79% in sample 2d-LML-02 to 52.94% in sample 0w-LML-02, with an average read duplication rate of 47.25%. After trimming low quality bases and removing adapter sequences, the number of reads and the average read length decreased to about 82 million reads per sample and an expected average read length of 23 nt for both R1 and R2 reads. For all samples about 62% of the reads could be assigned to annotated features by using featureCounts and including multimapping reads (between 58%–68%). On average, 15% of the reads could be mapped unambiguously onto the genome into regions with no annotated features, while about 23% of the reads could not be mapped unambiguously onto the rat genome (either reads not mapping to the reference genome at all or reads mapping to multiple features). Up to 2.69% of the unprocessed reads could be aligned to rRNA based on the sortMeRNA default database with an average of 1.33% aligning to rRNA over all small RNA samples.

### 3.3 Usage of the presented data report

The dataset from our study offers a valuable resource for understanding the quantitative relationship between hepatic perfusion and function in the transcriptomes of healthy and venous-ligated livers. It enables comprehensive analyses not only on mRNAs but also on lncRNAs and miRNAs. Given the limited knowledge about lncRNAs and the lack of general computer programs for their identification, including essential secondary structure information and protein interactions, this dataset is particularly significant. It serves as a potential source for marker genes related to regeneration capacity and potential therapeutic targets. More specifically, the reader may systematically investigate the following: 1) Description of the liver-specific ncRNA landscape; 2) Comparison of ncRNAs and isoforms in different conditions of the liver: healthy and after portal vein ligation; 3) Comparison of ncRNAs and isoforms at different time points after ligation of the liver lobe.

## Data Availability

The datasets presented in this study can be found in online repositories. The names of the repository and accession number can be found below: https://www.ebi.ac.uk/ena, PRJEB74462. The assignment of samples to accession IDs is listed in Supplementary Data Sheet 2.csv.

## Footnotes

1
http://rfam.sanger.ac.uk

2
https://www.gencodegenes.org/human/

3
https://www.bioinformatics.babraham.ac.uk/projects/fastqc

4
https://ftp.ensembl.org/pub/release-108/fasta/rattus_norvegicus/dna/Rattus_norvegicus.mRatBN7.2.dna.toplevel.fa.gz

5
https://ftp.ensembl.org/pub/release-108/gtf/rattus_norvegicus/Rattus_norvegicus.mRatBN7.2.108.chr.gtf.gz

6
https://ftp.ncbi.nlm.nih.gov/refseq/R_norvegicus/annotation_releases/current/108/GCF_015227675.2_mRatBN7.2/GCF_015227675.2_mRatBN7.2_genomic.fna.gz

7
https://ftp.ncbi.nlm.nih.gov/refseq/R_norvegicus/annotation_releases/current/108/GCF_015227675.2_mRatBN7.2/GCF_015227675.2_mRatBN7.2_genomic.gtf.gz
